# Antiparasitic Activity and Essential Oil Chemical Analysis of the *Piper Tuberculatum* Jacq Fruit

**Published:** 2018

**Authors:** Valterlúcio dos Santos Sales, Álefe Brito Monteiro, Gyllyandeson de Araújo Delmondes, Emmily Petícia do Nascimento, Francisco Rodolpho Sobreira Dantas Nóbrega de Figuêiredo, Cristina Kelly de Souza Rodrigues, Josefa Fernanda Evangelista de Lacerda, Cícera Norma Fernandes, Maysa de Oliveira Barbosa, Adamo Xenofonte Brasil, Saulo Relison Tintino, Maria Celeste Vega Gomez, Cathia Coronel, Henrique Douglas Melo Coutinho, José Galberto Martins da Costa, Cícero Francisco Bezerra Felipe, Irwin Rose Alencar de Menezes, Marta Regina Kerntopf

**Affiliations:** a *Department of Biological Chemistry, Regional University of Cariri, Crato, CE, Brazil. *; b *Center for the Development of Scientific Research, Foundation Moisés Bertoni/Laboratories Diaz Gill, Asunción-Paraguay.*; c *Department of Molecular Biology, Federal University of Paraíba, Paraíba, PB, Brazil.*

**Keywords:** Antiparasitic activity, *Piper tuberculatum* Jacq, Essential oil, Terpenes, Natural products

## Abstract

With the increase of neglected diseases such as leishmaniasis and Chagas disease, there was a need for the search for new therapeutic alternatives that reduce the harm caused by medicine available for treatment. Thus, this study was performed to investigate the antiparasitic activity of the essential oil from the fruits of *Piper tuberculatum* Jacq, against lines of *Leishmania braziliensis* (MHOM/CO/88/UA301), *Leishmania infantum* (MHOM/ES/92/BCN83) and *Trypanosoma cruzi* (LC-B5 clone). Before running protocols, an analysis of the chemical composition of essential oil was conducted, which presented monoterpenes and sesquiterpenes. As major constituents, β-pinene and α-pinene were identified. Regarding to antiparasitic activity, the essential oil had an EC_50 _values of 133.97 µg/mL and 143.59 µg/mL against variations promastigotes of *L. infantum* and *L. braziliensis*, respectively. As for trypanocidal activity, the oil showed EC_50_ value of 140.31 µg/mL against epimastigote form of *T. cruzi*. Moreover, it showed moderate cytotoxicity in fibroblasts with LC_50_ value of 204.71 µg/mL. The observed effect may be related to the presence of terpenes contained in the essential oil, since it has its antiparasitic activity proven in the literature.

## Introduction

Leishmaniasis caused by protozoa of the *Leishmania* genus, one of the six infectious and parasitic diseases of major importance in the world, is endemic in 98 countries, present on four continents: Africa, America, Asia and Europe, with annual record of 1 million to 1.5 million cases (1). Every year about 2 million new cases are notified, having a high detection rate and ability to produce deformities (2).

This disease occurs in 12 countries in Latin America, 90% of cases occurring in Brazil, especially in the Northeast region. In the country, the disease is found throughout the Brazilian territory except for the Southern region. Over the last 10 years, the annual average is of 3,156 cases, with an incidence of 2/100,000 cases per inhabitant. It is most common in children under 10 years old, and proportionally males are most affected (2).

Another annoyance disease neglected is Chagas, which began millions of years ago as enzootic diseases of wild animals, and subsequently transmitted to human beings characterized as an anthropozoonosis. Chagas disease is caused by the protozoan *T. cruzi*, which has affected about 12 million people only in Latin America and 75 million people are likely to acquire it (3).

Pharmacotherapy for leishmaniasis treatment and Chagas disease is a little bit scarce (4). This is due to commercial disinterest reflected by the fact that the parasitic diseases mainly affect developing countries, where the population has low purchasing power, leading to a low yield for factories, because the drugs would have to be affordable (5).

Chemotherapy with pentavalent antimony is widely used, but it is not free of side effects, requiring parenteral administration, extensive treatment and the emergence of resistance, justifying the search for effective alternative drugs, where medicinal plants are highlighted growing (6, 7). Based on this reasoning, the World Health Organization (WHO) emphasizes the urgent need to develop new medicine for the prophylaxis of these diseases (8).

In popular medicine, some species of the *Piper* genus showed antiparasitic activity; therefore may be required for employment as an alternative therapy (6). In developed studies, many compounds derived from *Piper* species have proven their parasitic activities (4, 6). The *Piper betle* extract showed activity against promastigotes of *Leishmania donovani* (9), *Piper chaba* was effective against promastigotes of *L. donovani* varieties (10), *Piper hispidum* was effective against amastigote form of *L. amazonensis* (11), and the extracts and fractions of *Piper ovatum* showed activity against the promastigote and amastigote varieties of *L. amazonensis* (12). Extracts and fractions of *Piper reginelli* (Mic) DC. var pallescens (C. DC.) Yunck were effective against promastigote form of *L. amazonensis* (13).

In this way, it is believed that *P. tuberculatum* Jacq. has the potential to combat parasitic diseases, by their kinship degree with the above species. *P. tuberculatum* Jacq. is a Piperaceae of economic and medicinal importance, popularly known as “monkey pepper”, found in regions of the states: Amazonas, Rondônia, Pará, Maranhão, Piauí, Ceará, Paraíba, Pernambuco, Rio de Janeiro and Mato Grosso. It is used empirically as sedative antidote for snake venom (14) and in the treatment of stomach disorders (15).

In this study, the objective was to assess the possible anti-parasitic effect of the essential oil from the fruits of *P. tuberculatum* Jacq., against lines of *L. braziliensis *and *L. infantum* as well as *T. cruzi*, contributing to the search for therapeutic alternatives for these diseases.

## Experimental

*Plant material and obtaining the essential oil*

The plant material (fruits of *P. tuberculatum* Jacq.) was collected at the farm Arajara in the city of Barbalha, State of Ceará, Brazil. The botanical identification was carried out by Professor Dr. Maria Arlene Pessoa da Silva and a voucher specimen was deposited in the Herbarium Caririense Dárdano de Andrade Lima - HCDAL of the Regional University of Cariri - URCA, cataloged under the registration number 10631.

Fresh fruits (3852 g) were subjected to hydrodistillation in a Clevenger type device. The material was weighed and placed in a glass flask, added distilled water and subjected to boiling for 2 h. At the end of that period, the extracted essential oil was treated with anhydrous sodium sulphate to eliminate the residual moisture.

*Chemical analysis of the essential oil*

Analysis of the chemical composition of the essential oil was performed by gas chromatography coupled to mass spectrometer (CG/EM Shimadzu model QP5050A) and provided with a capillary column DB-5HT of fused silica with 30 m long, 0.25 mm internal diameter, and film of 0.25 μm, with helium as carrier gas and flow 0.8 mL/min. The injector temperature was 250 °C and the detector temperature (or interface) was 200 °C. The column temperature was programmed from 35 °C to 180 °C at 4 °C/min and then 180 °C to 250 °C at 10 °C/min. The mass spectra were recorded from 30-450 *m/z*.

The individual components were identified by matching their mass spectra with 70 eV impact energy, with the data base using the library built through the spectrometer (Wiley, 229) and other two computers using the retention indices as a pre-selection (16, 17), as well as by visual comparison of fragmentation pattern with those reported in the literature (18, 19).

*Cell lines used*

For the *in-vitro* tests for *T. cruzi*, CL-B5 clone was used (20). The parasites stably transected with the gene for β-galactosidase of *Escherichia coli* (*lacZ*) were provided by Dr. F. Buckner through Commemorative Gorgas Institute (Panama). Epimastigotes forms were cultured at 28 °C in *Liver Infusion Tryptose Broth* (Difco, Detroit, MI) supplemented with 10% Fetal Bovine Serum (FBS) (Gibco, Carlsbad, CA), penicillin (Ern, SA, Barcelona, Spain) and streptomycin (Reig Jofr SA, Barcelona, Spain) as described by Le Senne *et al*., (21). The cells were collected for testing in the exponential phase of growth.

Culture of *Leishmania* spp. were obtained from the Health Science Research Institute, Asunción, Paraguay - IICS and identified by isoenzyme analysis. The maintenance of lines, forms of cultivation and isolation of promatigotas forms of *Leishmania* spp. followed procedures described by Roldos *et al*., (22). The inhibitory action of these promastigotes forms were performed using the *L. braziliensis* (MHOM / CO / 88 / UA301) and *L. infantum* lines (MHOM/ES/92/BCN83), cultured at 22 °C in Schneider’s Drosophila supplemented with 20% FBS.

Cytotoxicity assays used NCTC929 fibroblasts lines were grown in Minimal Essential Medium (Sigma). The culture medium was supplemented with heat inactivated FBS (10%), penicillin G (100 U/mL) and streptomycin (100 mg/mL). Cultures were maintained at 37 °C in a humidified atmosphere with 5% CO_2_. The viability of the lines was evaluated through the use of resazurin as a colorimetric method (23).

*Reagents*

The sodium resazurin was obtained from Sigma-Aldrich (St. Louis, MO) and stored at 4 °C protected from light and prepared with 1% phosphate buffer, pH 7 and filter sterilized before use. The red-β-D-galactopyranoside chlorophenol (CPRG, Roche, Indianapolis, IN) was dissolved in a solution of Triton X-100 0.9% (pH 7.4). Penicillin G (Ern, SA, Barcelona, Spain), streptomycin (Reig Jofré SA, Barcelona, Spain) and Dimethyl sulfoxide (DMSO) were also used.

*Anti-epimastigote activity test*

The test was performed in microplates with 96 cavities with cultures in exponential phase, as described by Vega *et al*., (24). The epimastigotes were inoculated at a concentration of 1 x 105 mL-1 in 200 μL of tryptose liver broth. The plates were then incubated with the drugs at concentrations of 100 and 500 μg/mL at 28 °C for 72 h. After this time, 50 μL of CPRG solution was added to achieve a final concentration of 200 μM. The plates were incubated for an additional period of 6 h at 37 °C and were subjected for visualization under 595 nm. Each experiment was carried out twice and independently, with each concentration triplicate tested in each experiment. The efficiency of each compound was estimated by calculating the percentage of anti-epimastigotes activity (EA%).

*Anti-promastigote activity test*

Cultures of promastigotes forms of *L. braziliensis *and* L. infantum* were grown to a concentration of 10^6^ cells/mL and then transferred to the test. The compounds were dissolved in DMSO to the concentrations to be tested, and transferred to the microplates. Each assay was triplicate performed. The activity of the compounds was assessed after 72 h by direct counting of the cells after serial dilutions and compared to an untreated control.

*Cytotoxicity test*

NCTC929 fibroblasts were plated in microdilution plates of 96 cavities at a final concentration of 3 × 10^4^ cells/cavity. The cells were cultured at 37 °C in atmosphere with 5% CO_2_. After that, the culture medium was removed and the compounds were added 200 μL, being carried out a new culture for 24 h. After this incubation, 20 μL of a 2 mM solution of Resazurin was added to each cavity. The plates were incubated for 3 h and the reduction of resazurin was determined by absorbance at dual wavelengths of 490 and 595 nm. The control value (blank) was subtracted.

*Statistical analysis*

To determine the LC_50_ and EC_50_ data, they were analyzed using PROBITOS software.

## Results and Discussion

The use of essential oils in traditional medicine has grown over the years, based on their medicinal activities, such as bactericidal, fungicidal, virucidal, anti-inflammatory, antispasmodic effects, among others. Essential oils are derived from aromatic plants and consist of secondary metabolites (25). A pharmacological property reported in the literature, being used as an alternative in antiparasitic therapy, since the increase of resistant parasites to drugs available in the market, directing for the encouragement of the development of research, by the search for new therapeutic agents (7, 26).

In the investigation of chemical composition of essential oil *P. tuberculatum* (EOPT), 0.34% yield is observed, being possible to identify 98.97% of constituents as shown in Table 1. Out of these identified compounds, there was emphasis on the β-pinene (27.74%) and α-pinene (26.54%), major compounds.

Among the chemicals already identified in species of *Piper*, several terpenes have already been described in the literature (27). In chemical analysis of essential oil of *Piper aduncum*,* Piper amalago*,* Piper arboreum*,* Piper cernuum*,* Piper hispidum*,* Piper regnelii*,* Piper submarginalum*,* Piper vicosanum *e* Pothomorphe umbellata*, os principais monoterpenos encontrados foram α-pineno, β-pineno, espatulenol, E-carioﬁleno, óxido de carioﬁleno, germacreno D e limoneno (28).

Terpenes identified in EOPT are shown into two types, sesquiterpenes and monoterpenes. The major components are structural isomers, being able to differentiate toxicity and types of biological activity (29). Besides them, it was also detected β-caryophyllene, β-ocimene, myrcene, limonene and other constituents in minor amounts.

**Table 1:** Chemical composition of the essential oil of *Piper tuberculatum* Jacq

**Components**	**(%)**	**TR (min)**	**Kovats indices**
α-pinene	26.54	12.54	939
Sabinene	2.65	14.67	976
β-pinene	27.74	14.94	980
Myrcene	1.55	15.53	991
Limonene	3.02	17.86	1031
1,8-cineole	1.41	18.05	991
β–ocimene	12.45	18.92	1040
α-terpineol	0.81	27.70	1189
α-copaene	1.29	38.55	1376
β-caryophyllene	14.38	41.13	1418
α-humulene	1.26	43.22	1455
Germacrene D	1.09	45.10	1480
Nerolidol	0.94	47.37	1534
Spathulenol	1.02	47.86	1576
Caryophyllene oxide	2.82	47.97	1581
TOTAL	98.97		

**Table 2:** Antiparasitic and cytotoxicity activity of the essential oil of *Piper tuberculatum*

**Drug**	**Concentration ** **(µg/mL)**	**Cytotoxicity activity**		**Antiparasitic activity**					
		**%CTF**	**LC**_50_ **(µg/mL)**	***T. cruzi*** **(%AE)**	**EC**_50_ **(µg/mL)**	***L. infantum*** **(%AP)**	**EC**_50_ **(µg/mL)**	***L. braziliensis*** **(%AP)**	**EC**_50_ **(µg/mL)**
EOPT	1000	100	204.71	70.00	140.31	100	133.97	100	143,59
	500	98.19		70.00		100		86.53	
	250	82.17		70.41		100		71.70	
	125	1.75		52.96		50.50		51.01	
NIFU	1	-	-	54.9	0.91	-	-	-	-
	0,5	-		45.6		-		-	
PENTA	6.25	-	-	-	-	54.2	5.69	-	-
	3.125	-		-		15.5		-	
METRO	2	-	-	-	-	-	-	100	0.51
	1	-		-		-		97.9	

EOPT: Essential oil of *Piper tuberculatum*; NIFU: Nifurtimox; PENTA: Pentamidine; METRO: Metronidazole; AP: Anti-promastigote activity; AE: Anti-epimastigote activity; CTF: Cytotoxicity in Fibroblasts.

As their antiparasitic activity, EOPT caused death percentage of > 87% against the parasitic forms of *L. infantum* of the studied concentrations. Before the promastigote variation of *L. infantum* in the concentration of 125 μg/mL, it caused the death of 50.5% of parasites. The anti-promastigote percentage against *L. braziliensis* was > 77% in the tested concentrations. Furthermore, EOPT in the 125 μg/mL concentration caused a parasite death of 51%. Against *T. cruzi*, the essential oil had, overall, an activity of 65% where the 125 μg/mL concentration was effective in producing inhibition of 52.9% (Table 2).

In the cytotoxic activity, OEPT caused mortality of 1.75%, 87.12%, 98.19% and 100% of fibroblasts at concentrations of 125, 250, 500 and 1000 μg/mL. Concentration of 125 μg/mL showed a low toxicity when compared to other concentrations with a cytotoxic percentage of 1.75% (Table 2).

In the literature, many *Piper* species have been described with antiparasitic effect. This is the case of the essential oil of *Piper bredermayeri*, *Piper cf. divaricatum, Piper. var brachypodom*, which showed activity against epimastigote forms of *T. cruzi* and promastigotes of *L. infantum* (30). *Piper auritum* showed to be active against a variety of *L. braziliensis* promastigote (31), as well as the *Piper claussenianum* was effective against variety of *L. amazonensis* promastigote (32) and *Piper malacophyllum* was effective against *T. cruzi* and *L. braziliensis* (33).

Some chemical compounds such as propanoic acid, esters, lignans, amides and terpenes, probably are responsible for the effect anti-leshmanicidal already identified in *Piper* species (32, 34, 35 and 36).

Such compounds may be associated with the effects observed in this study, since the main constituent of α-pinene of *P. bredermeyeri* and *P. cf. divaricatum*, same genus species of *P. tuberculatum* Jacq, was considered responsible for the effect against epimastigotes and amastigotes of *T. Cruzi* and promastigotes of* L. infantum*. (30). Other studies that have investigated this monoterpene alone showed significant results, justifying the effect obtained when using EOPT rich in α pinene. In the study of Sobral-Souza *et al*., (37) the α-pinene in the concentration of 100 µg/mL was effective against strains of the parasite *L. braziliensis,* corroborating the results found in this article.

Other compounds in EOPT were also tested against *Leishmania* spp. strains. Limonene also present in EOPT showed an EC_50_ of 252.6 µM/mL against epimastigote and promastigote of *L. braziliensis* variations (38). In addition, Myrcene obtained from *Cymbopogon citratus* showed an EC_50_ of 164 µg/mL against cultures of promastigotes of *L. infantum* (39). Izumi (40) noted that β-caryophyllene was effective against species of *Leishmania *spp. showing a synergy when combined with copalic acid present in the essential oil of copaiba. This study helps to prove that the terpenes may have synergistic anti-parasitic activity, a finding that is quoted on some articles, though not proven.

Limonene in its isomeric forms was effective to reduce the number of strains of *T. cruzi* epimastigote where the R-limonene showed a lower EC_50_ than S-limonene, showing greater efficiency (41). The caryophyllene obtained an EC_50_ of 30 µg/mL and 100 µg/mL against epimastigote and promastigotes variations of *T. cruzi *and *L. braziliensis* respectively (42).

Essential oils did not obtain their widely action mechanisms elucidated. Probably its lipid solubility and its secondary constituents influence in their antiparasitic activity, since it allows entry into cell membranes regulating structures of different layers of phospholipids, causing cellular damage (25).

Some compounds derived from essential oils showed different mechanism of action. β-caryophyllene produced disorganization of kinetoplast, forming concentric membranous vacuoles, lipid peroxidation, and changes in cell membrane integrity (40). This same compound is present in EOPT, suggesting that these EOPT may also have mechanisms of action. Other terpenes such as citral, presented antiparasitic activity against promastigotes of *L. amazonensis* species by modifying the morphology and ultrastructure of the parasite, producing mitochondrial swelling, two flagella and exocytic projections of the flagellar bag (43). The linalool also present in terpenes class produced significant changes of mitochondrial cristae parasites (44).

For the treatment of some tropical diseases, medicinal plants have been shown to be a viable source in the search for new alternatives (31). Thus, this study showed that the essential oil obtained from the fruits of *P. tuberculatum* Jacq., had good antiparasitic potential, corroborating some data already described in the literature and that probably the major compounds are responsible for the observed effect. Nevertheless, it is necessary further testing in order to elucidate its mechanism of action.
